# Anti-Neuroinflammatory effects of the extract of *Achillea fragrantissima*

**DOI:** 10.1186/1472-6882-11-98

**Published:** 2011-10-21

**Authors:** Anat Elmann, Sharon Mordechay, Hilla Erlank, Alona Telerman, Miriam Rindner, Rivka Ofir

**Affiliations:** 1 Department of Food Quality and Safety, Volcani Center, Agricultural Research Organization, Bet Dagan, 50250, Israel; 2 Dead Sea & Arava Science Center and Department of Microbiology & Immunology, Ben-Gurion University of the Negev, Beer-Sheva, 84105, Israel

**Keywords:** *Achillea fragrantissima*, microglial cells, neuroinflammation, nitric oxide, matrix metalloproteinase-9, cyclooxygenase-2

## Abstract

**Background:**

The neuroinflammatory process plays a central role in the initiation and progression of neurodegenerative diseases such as Parkinson's and Alzheimer's diseases, and involves the activation of brain microglial cells. During the neuroinflammatory process, microglial cells release proinflammatory mediators such as cytokines, matrix metalloproteinases (MMP), Reactive oxygen species (ROS) and nitric oxide (NO). In the present study, extracts from 66 different desert plants were tested for their effect on lipopolysaccharide (LPS) - induced production of NO by primary microglial cells. The extract of *Achillea fragrantissima *(*Af*), which is a desert plant that has been used for many years in traditional medicine for the treatment of various diseases, was the most efficient extract, and was further studied for additional anti-neuroinflammatory effects in these cells.

**Methods:**

In the present study, the ethanolic extract prepared from *Af *was tested for its anti-inflammatory effects on lipopolysaccharide (LPS)-activated primary cultures of brain microglial cells. The levels of the proinflammatory cytokines interleukin1β (IL-1β) and tumor necrosis factor-α (TNFα) secreted by the cells were determined by reverse transcriptase-PCR and Enzyme-linked immunosorbent assay (ELISA), respectively. NO levels secreted by the activate cells were measured using Griess reagent, ROS levels were measured by 2'7'-dichlorofluorescein diacetate (DCF-DA), MMP-9 activity was measured using gel zymography, and the protein levels of the proinflammatory enzymes cyclooxygenase-2 (COX-2) and induced nitric oxide synthase (iNOS) were measured by Western blot analysis. Cell viability was assessed using Lactate dehydrogenase (LDH) activity in the media conditioned by the cells or by the crystal violet cell staining.

**Results:**

We have found that out of the 66 desert plants tested, the extract of *Af *was the most efficient extract and inhibited ~70% of the NO produced by the LPS-activated microglial cells, without affecting cell viability. In addition, this extract inhibited the LPS - elicited expression of the proinflammatory mediators IL-1β, TNFα, MMP-9, COX-2 and iNOS in these cells.

**Conclusions:**

Thus, phytochemicals present in the *Af *extract could be beneficial in preventing/treating neurodegenerative diseases in which neuroinflammation is part of the pathophysiology.

## Background

The increase in the life span of populations in the Western world has been accompanied by an elevation in the frequencies of neurodegenerative diseases, e.g., Alzheimer's and Parkinson's diseases. In these diseases, a gradual and progressive neuronal cell death occurs, amongst other, as a consequence of increased nitrosative and oxidative stress and an uncontrolled neuroinflammatory response [1-3]. These processes play a pivotal role in the initiation and progression of various neurodegenerative diseases and involve the activation of microglial cells [4]. Microglial cells, are cells of the macrophage lineage in the central nervous system (CNS), and are quiescent in the normal brain. However, they can be activated by the cytokines produced by infiltrating immune effector cells in response to CNS injury or to the lipopolysaccharide (LPS) excreted during bacterial infection. Activated microglial cells release either neurotrophic factors, supporting neuronal cell survival, or neurotoxic factors, such as oxygen radicals, nitric oxide (NO) and proinflammatory cytokines [4]. While microglial activation is necessary and critical for host defense, prolonged and excessive stimulation of these cells initiates an inflammatory cascade in the CNS that contributes to the pathogenesis of several neurodegenerative diseases. Therefore, controlling microglial activation is regarded as a promising therapeutic target to combat neurodegenerative diseases.

Cyclooxygenase-2 (COX-2) and induced NO-synthase (iNOS) are inducible forms of enzymes which are up regulated in activated microglia in response to inflammatory challenge. The induction and regulation of these enzymes are tightly coupled and thought to contribute to the pathogenesis of various diseases, including neurodegenerative diseases [5]. The excessive amounts of NO, a free radical produced by iNOS, and of prostaglandin E, an arachidonic acid metabolite produced by COX-2, which are secreted by activated microglial cells during the neuroinflammatory process, cause nitrosative stress and brain cell death [5,6]. NO is a free radical, and high levels of NO have been implicated in the pathogenesis of stroke, trauma, demyelinating, and neurodegenerative diseases [7]. iNOS and COX-2 are upregulated in activated microglia in response to inflammatory stimuli such as Alzheimer's amyloid peptide, interferon gamma (IFNγ) and bacterial LPS. Co-induction and co-regulation of iNOS and COX-2 have also been demonstrated in a number of cell culture studies and in inflammatory animal model systems [8].

Other molecules that are secreted by stimulated microglial cells include tumor necrosis factor alpha (TNFα) and interleukin 1β (IL-1β) [4], both of which can cause neuronal cell death both directly and indirectly via the induction of NO and free radicals in microglial cells [9].

Matrix metalloproteinase-9 (MMP-9) is a zinc-dependent enzyme, that belongs to the family of MMPs and contributes to the neuroinflammatory response in neuroinflammation and in neurodegenerative diseases such as amyotrophic lateral sclerosis [10], and Alzheimer's disease [11,12]. MMP-9 is also upregulated in rodent models of cerebral ischemia, hemorrhage and trauma [13-15] and after its activation by proteases and ROS [16,17] can disrupt the blood brain barrier (BBB), a disruption that leads to extravasation of blood proteins, to brain edema, to cerebral hypoperfusion, and ultimately to neuronal damage [18-20]. A deleterious role for MMP-9 is indicated because MMP-9 knockout mice are protected against focal cerebral ischemia [21,22] brain trauma [23] and experimental encephalomyelitis [24]. Brain microglial cells and endothelial cells have been shown to be a source of MMP-9 [12]. Microglial cells serve as important source of MMP-9, and lipopolysaccharide (LPS), IL-1β and TNF-α were shown to stimulate its production from these cells [12].

Thus, elevated activity and/or expression of iNOS, COX-2, MMP-9, IL-1β and TNF-α in brain cells have been implicated in the cascade of events leading to neurodegenerative diseases.

Many herb and plant extracts are used as folk medicines for various kinds of inflammatory diseases, organ dysfunctions and systemic disorders. In the present study we screened ethanolic extracts prepared from 66 different desert plants for their capacity to inhibit NO production from LPS-activated microglial cells. The extract of *Achillea fragrantissima *(*Af; *Asteraceae) exerted the most potent inhibitory activity.

*Achillea fragrantissima *(*Af*) is a desert plant that has been used for many years in traditional medicine in the Arabia region for the treatment of respiratory diseases and gastrointestinal disturbances [25-28]. It was therefore thought worthwhile to investigate the effects of *Af *on neurodegenerative diseases, effects that have not been studied to date. The present study describes the anti-neuroinflammatory activities of this plant.

## Methods

### Reagents

Dulbecco's modified Eagle's medium (DMEM), RPMI-1640 (with or without phenol red), Leibovitz-15 medium, glutamine, antibiotics (10,000 IU/ml penicillin and 10,000 μg/ml streptomycin), soybean trypsin inhibitor, fetal bovine serum (FBS) and Dulbecco's phosphate buffered saline (PBS) (without calcium and magnesium) were purchased from Biological Industries (Beit Haemek, Israel); Griess reagent and rabbit anti COX-2 polyclonal antibody were obtained from Cayman chemical, Ml, USA; DreamTaq Green PCR master Mix (2x) and ReverAid First Strand cDNA Synthesis Kit were purchased from Fermentas life sciences (Eisenberg Bros. Ltd, Israel). iNOS polyclonal antibody was purchased from AbD Serotec, Ox, UK; Horseradish peroxidase (HRP)-conjugated anti-rabbit IgG was obtained from Jackson ImmunoResearch Laboratories Inc. Baltimore, USA; Monoclonal mouse anti-β-actin was purchased from MP Biomedicals, Ohio, USA; LPS (*Escherichia coli *0127 B:8), 2-mercaptoethanol, L-NMMA (N^G^-Methyl-L-arginine acetate salt), Gelatin, Crystal violet and protease inhibitor cocktail were purchased from Sigma Chemical Co. (St Louis, MO, USA). 2,2'-Azobis(amidinopropane) (ABAP) was obtained from Wako chemicals (Richmond, VA), and 2'7'-dichlorofluorescein diacetate (DCF) were purchased from Sigma Chemical Co. (St Louis, MO, USA).

### Preparation of Plant Extracts

The plants were collected in the Arava Valley, and the voucher specimens have been kept and authenticated as part of the Arava Rift Valley Plant Collection; VPC (Dead Sea & Arava Science Center, Central Arava Branch, Israel, http://www.deadseaarava-rd.co.il/_Uploads/dbsAttachedFiles/Arava_Rift_Valley_Plant_Collection.xls) under the accession code AVPC0040. Freshly collected plants were dried at 40°C for three days and extracted in ethanol (96%). The liquid phase was then evaporated off, and the dry material was dissolved in DMSO to a concentration of 100 mg/mL to produce the various extracts, including *Af *extract.

### Preparation of Primary Microglial Cell Cultures

Cultures of primary rat microglial cells were prepared from cerebral cortices of 1- to 2-day-old neonatal Wistar rats as described [29]. The research was conducted in accordance with the internationally accepted principles for laboratory animal use and care, as found in the US guidelines, and was approved by the Institutional Animal Care and Use Committee of The Volcani Center, Agricultural Research Organization.

### Nitrite Quantification

For NO measurements, 1 × 10^5 ^microglial cells/well were plated in a 24-well tissue culture plate. After 36 h of incubation in RPMI-1640 (without phenol red), containing 2% FBS, 2 mM glutamine, 100 U/mL penicilin, 100 μg/mL streptomycin, 1 mM sodium pyruvate, and 50 μM β-mercaptoethanol, cells were stimulated with LPS (4.5 ng/mL). NO levels in the culture medium were estimated by measuring the concentration of nitrite, its stable metabolite, with Griess reagent as described [29]. Fresh culture medium was used as the blank in all the experiments.

### Determination of Cell Viability

Cell viability was determined using a commercial colorimetric assay (Roche Applied Science, Germany), based on the measurement of lactate dehydrogenase (LDH) activity released from the cytosol of damaged cells into the supernatant, according to the manufacturer's instructions. In MMP-9 assay, cell viability was determined by a modification of the crystal violet assay [30]. At the end of cell treatments, cells were fixed with 150 μL of 5% (v/v) formaldehyde (in PBS) for 15 min at room temperature. Plates were washed by submersion in de-ionized water, dried and stained for 15 min with 150 μL of a 1% crystal violet solution. After careful aspiration of the crystal violet solution the plates were washed with de-ionized water, and dried prior to the solubilization of the bound dye with 150 μL of a 33% aqueous glacial acetic acid solution. The optical density of the plates was measured at 540 nm (with a 690 nm reference filter) in a microplate spectrophotometer.

### Western Blot Analysis

Microglial cells were plated at a concentration of 4 × 10^6^/10 mL and treated as described above. Following treatment, the cells were processed and subjected to Western blot analysis as described [29].

### Measurement of TNFα Levels in Conditioned Media

For TNFα measurements, 3.5 × 10^4 ^cells/well were plated on a 24-well tissue culture plate. After 24 h of incubation in DMEM containing 10% FBS, cells were stimulated with LPS (100 ng/mL). Five hours later, conditioned media from duplicate wells per sample were collected and tested for cytokine levels with a rat TNFα ELISA kit (Diaclone^®^; Gen-Probe Life Sciences Ltd. France), used according to the manufacturer's instructions.

### Cellular Antioxidant Activity of *Af* Extract

Intracellular ROS production was detected using the non-fluorescent cell permeating compound, 2'7'-dichlorofluorescein diacetate (DCF-DA). DCF-DA is hydrolyzed by intracellular esterases and then oxidized by ROS to a fluorescent compound 2'-7'-DCF. Peroxyl radicals are generated by thermolysis of 2,2'-Azobis(amidinopropane) (ABAP) at physiological temperature. ABAP decomposes at approximately 1.36 × 10^-6^s^-1 ^at 37°C, producing at most 1 × 10^12 ^radicals/ml/s [31-33]. Microglial cells were plated in DMEM containing 2% FBS, 2 mM glutamine, 100 U/mL penicillin and 100 μg/mL streptomycin, onto 24 wells plates (300,000 cells/well) and were incubated for 1 hr with *Af *extract. Then microglial cells were preloaded with DCF-DA for 30 min, washed twice with PBS, and ABAP (0.6 mM final concentration) was then added. The fluorescence, which indicates ROS levels, was measured in a plate reader with excitation at 485 nm and emission at 520 nm.

### Determination of MMP Activities in Conditioned Media of Microglial Cells

MMP-9 was quantified by gelatin zymography [18]. For the determination of MMP activities in conditioned media of microglial cells, 1 × 10^5 ^cells/well were plated in a 24-well tissue culture plate in (DMEM containing 2 mM glutamine, 100 U/mL penicilin, and 100 μg/mL streptomycin). After 24 h of incubation the medium was replaced with fresh medium and cells were stimulated with LPS (4.5 ng/mL). The medium conditioned by the cells was collected 24 h after an LPS challenge, and was concentrated x3. Samples (21 μL) of CM were mixed with non-reduced sample buffer and were loaded on 8% SDS-polyacrylamide gels (SDS-PAGE) that contains 1 mg/mL gelatin type A. Electrophoresis was performed under non-reducing conditions. Gels were washed (30 min) in 2.5% Triton X-100 to remove SDS and then for 30 min in reaction buffer (50 mM Tris-HCl, pH 7.5, 0.02% Brij 35, 10 mM CaCl_2_, 200 mM NaCl). The reaction buffer was then changed to a fresh one and the gels were incubated (24 h, 37°C) in a shaking incubator. Gelatinolytic activity was visualized by staining the gels with 0.5% Coomassie brilliant blue. The densities of the specific protein bands were quantified by the ImajJ image analysis and processing program.

### RNA Extraction and Two-Step RT-PCR

RNA was prepared using TRI reagent (Molecular Research Center, Inc., Cincinnati, OH) according to manufacturer instructions. Genomic DNA was removed from the RNA samples by using 50 units of RNase-free DNaseI at 37°C for 1 h. For cDNA preparation, RNA (20 μg) was incubated with reverse transcriptase and Oligo (dT) 18 primer (0.5 μg/uL) for 1 h at 42°C followed by 10 min at 72°C. For PCR, reaction mixture contained the following: 1 μL of cDNA, 100 ng of each primer, 12.5 μL of DreamTaq PCR Mix (2X) and doubly distilled water to 20 μL. The following conditions were used for IL-1β and for the control gene β-actin: 5 min at 95°C; 30 s at 94°C, 30 s at 50°C and at 53°C, respectively, and 30 s at 72°C for 35 cycles and 25 cycles, respectively. Products were examined by agarose gel electrophoresis. The primers used were: IL-1β: 5'-TTGCCCGTGGAGCTTC-3' and 5'-CGGGTTCCATGGTGAAC-3'; α-tubulin: 5'-CTCCATCCTCACCACCCACAC-3' and 5'-CAGGGTCACATTTCACCATCT. The densities of the specific RNA bands were quantified by the ImajJ image processing and analysis in Java program.

### Data Analysis

Statistical analyses were performed with one-way ANOVA followed by Tukey-Kramer multiple comparison tests using Graph Pad InStat 3 for windows (GraphPad Software, San Diego, CA, USA).

## Results

### Extracts of various desert plants affect NO production by LPS-activated microglial cells

**Figure 1:**
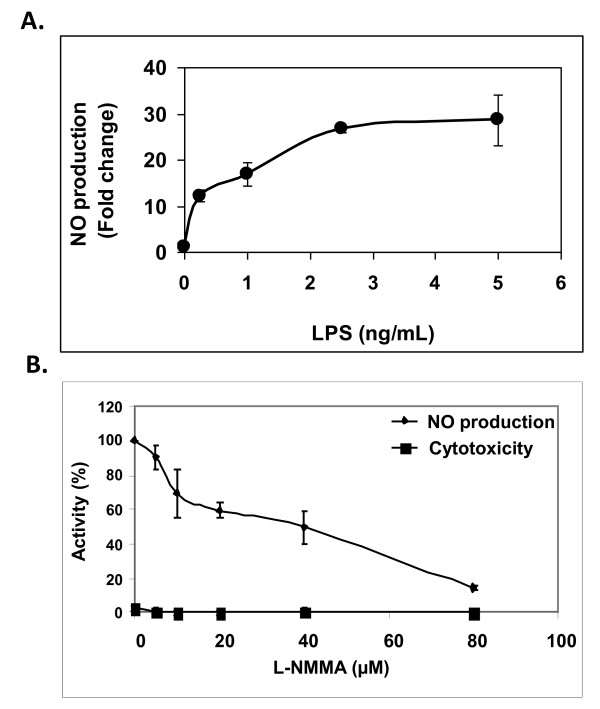
**NO production by LPS-activated primary microglial cells**. (A) Microglial cells were stimulated with different concentrations of LPS. (B) L-NMMA was added concomitant with activation by LPS. NO levels in cell conditioned supernatants were measured 20 h later. The results represent means ± SEM of 3 separate experiments (n = 9).

In order to conduct a first selection for prospective anti-neuroinflammatory activity, extracts from 66 different desert plants, which belong to 23 different plant families, were tested for their ability to down regulate NO production by activated microglial cells. For that purpose, we used a system in which stimulation of primary microglial cells with LPS induced significant increase of NO production (Figure 1A). Induction of NO production from LPS-activated microglial cells was specifically inhibited (90%) by L-NMMA (N^G^-Methyl-L-arginine acetate salt), a specific inhibitor of NOS (Figure 1B).

To exclude the possibility that reduction in NO secreted by microglial cells was due to the direct toxicity of the plant extracts to the cells, we tested cell toxicity by LDH release into culture media. For the 46 plants presented in Table 1 the extract-induced cytotoxicity was negligible at concentrations of 100 μg/mL used in this screening procedure (data not shown). Twenty plant extracts that exhibited cytotoxic effect to the microglial cells were excluded from the study. The distribution of the 46 non-toxic extracts according to their extent (%) of inhibition of NO release is presented in Table 1. It can be seen that 10 extracts (21% of the tested plants) upregulated NO production from activated microglial cells, and the other plant extracts inhibited the NO production to various degrees.

**Table 1:** The effect of different desert plant extracts on NO production by LPS-activated microglial cells ^a^.

Plant family	Plant Species	NO production (%) Mean ± SEM
Asteraceae	*Achillea fragrantissima*	30 ± 5 **
Papilionaceae	*Crotalaria algyptiaca*	63 ± 2 **
Polygonaceae	*Emex spinosa*	63 ± 6 **
Polygonaceae	*Rumex vesicarius*	64 ± 6 **
Capparaceae	*Cleome droserifolia*	66 ± 5 **
Asteraceae	*Launala nudicaulis*	69 ± 7 **
Amaranthaceae	*Aerva javanica*	71 ± 4 **
Asclepiadaceae	*Calotropis procera*	72 ± 7 **
Tamaricaceae	*Tamarix nilotica*	73 ± 6 **
Chenopodiaceae	*Hammada salicornica*	74 ± 6 **
Asclepiadaceae	*Pergularia tomentosa*	77 ± 4 *
Cruciferae	*Matthiola livida*	77 ± 8 *
Malvaceae	*Abutilon hirtum*	79 ± 8 *
Gramineae	*Schismus arabicus*	80 ± 8
Brassicaceae	*Moricandia nitens*	83 ± 6
Brassicaceae	*Brassica tournefortii *	84 ± 7
Asclepiadaceae	*Pentatropis nivalis*	84 ± 5
Gramineae	*Stipagrostis ciliate*	85 ± 3
Chenopodiaceae	*Salsola cyclophylla*	87 ± 4
Solanaceae	*Solanum incanum*	87 ± 4
Zygophyllaceae	*Tribulus bimucronatus*	88 ± 5
Chenopodiaceae	*Atriplex holocarpa*	89 ± 3
Molluginaceae	*Glinus lotoides*	92 ± 9
Tamaricaceae	*Tamarix aphylla*	94 ± 6
Chenopodiaceae	*Hammaola salicornica*	94 ± 3
Chenopodiaceae	*Salsola gaetula*	94 ± 8
Salvadoraceae	*Salvadora persica*	94 ± 6
Ephedraceae	*Ephedra aphylla*	96 ± 7
Chenopodiaceae	*Hammada scoparia*	96 ± 8
Solanaceae	*Lycium shawii*	97 ± 4
Chenopodiaceae	*Salsola cyclophylla*	97 ± 5
Capparaceae	*Capparis spinosa *	98 ± 7
Euphorbiaceae	*Ricinus communis*	99 ± 5
Thymelaeaceae	*Thymelaea hirsute*	99 ± 7
Apiaceae	*Deverra triradiata*	94 ± 7
Chenopodiaceae	*Salsola vermiculata*	100 ± 6
Gramineae	*Typha Domingensis*	101 ± 5
Chenopodiaceae	*Anabasis setifera*	102 ± 7
Gramineae	*Stipagrostis plumosa*	102 ± 4
Solanaceae	*Solanum nigrum*	109 ± 9
Chenopodiaceae	*Atriplex leucoclada*	113 ± 8
Mimosoideae	*Acacia raddiana*	114 ± 7
Mimosoideae	*Acacia tortilis*	117 ± 6
Zygophyllaceae	*Zygophyllum album*	140 ± 9 **
Gramineae	*Lasiurus scindicus*	157 ± 9 **
Plantaginaceae	*Plantago cylindrica*	157 ± 9 **

**^a ^**The results represent the mean ± SEM of 2-3 experiments (and of 8-9 experiments for *Af*) performed in triplicated or tetraplicates. The ethanolic extracts (100 μg/ml) were added to the microglial cells concomitant with the addition of LPS. * p < 0.05, ** p < 0.01

The extract of *Achilea fragrantissima *was the most efficient extract and inhibited ~70% of the NO released with respect to the LPS-activated cells. Therefore we have further characterized the anti-neuroinflammatory effects of this extract.

#### Attenuation by the *Af* Extract of NO release in LPS-Stimulated Microglial cells

**Figure 2:**
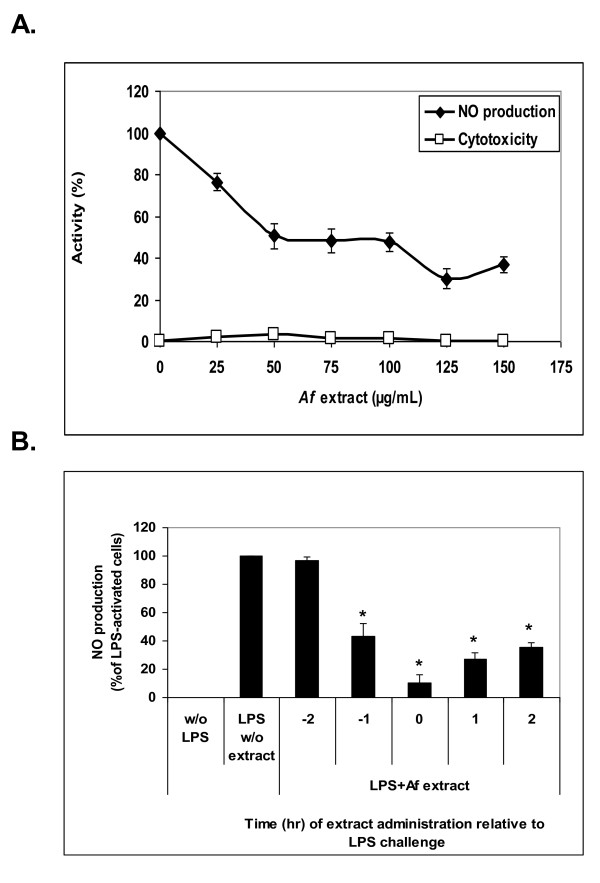
**Inhibition of NO production by activated microglial cells in response to *Af *extract**. (A) Microglial cells were treated with different concentrations of the extract and concomitantly activated by LPS (4.5 ng/mL) for 20 h. (B) *Af *extract was added before, concomitant with, or after activation by LPS (4.5 ng/mL). NO levels in cell conditioned supernatants were measured. The results represent means ± SEM of 3 separate experiments (**A**, n = 9) or 2 separate experiments (B, n = 6). * *p *< 0.01; ** *p *< 0.001.

**Figure 3:**
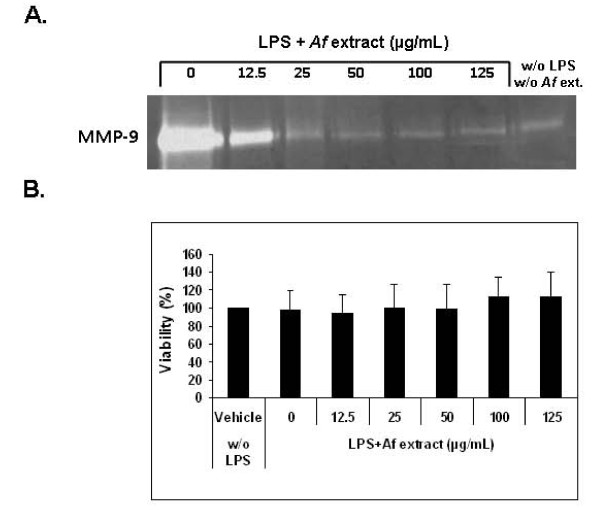
**Down-regulation of MMP-9 activity in activated microglial cells by *Af *extract**. Microglial cells were treated with the indicated concentrations of *Af *extract, followed by stimulation with LPS (4.5 ng/mL). After 24 h: A. conditioned media were collected and tested for MMP-9 activity by gel zymography. The zymogram represents two independent experiments. B. Cell viability was determined by the crystal violet assay. The histogram represents the means ± SD of two independent experiments (n = 2).

*Af *extract was tested for its ability to downregulate NO production from primary cultures of LPS-activated microglial cells. Figure 2A demonstrates that the LPS-elicited nitrite accumulation was markedly inhibited by the *Af *extract in a dose-dependent manner. To exclude the possibility that reduction in NO secreted by the activated microglial cells was due to the direct toxicity of the plant extract to the cells, we tested cell toxicity following treatment with *Af *extract by measuring Lactate dehydrogenase (LDH) release into culture media. The LDH assay showed that the extract-induced cytotoxicity was negligible at concentrations below 150 μg/mL. Viability of cells was tested also by the crystal violet assay and showed similar results (Figure 3B). To elucidate the optimal time for the addition of *Af *extract with respect to LPS addition, three different regimes were tested: the cells were pre-incubated in the presence of the extract for 1 or 2 h before the addition of LPS; the extract was added concomitantly with LPS; or the extract was added 1 or 2 h after cell activation. The most effective times for the addition of *Af *extract were concomitant with or after cell stimulation (Figure 2B).

### *Af *extract reduces 2,2'-azobis(amidinopropane) (ABAP)-mediated peroxyl radicals levels in microglial cells

**Figure 4:**
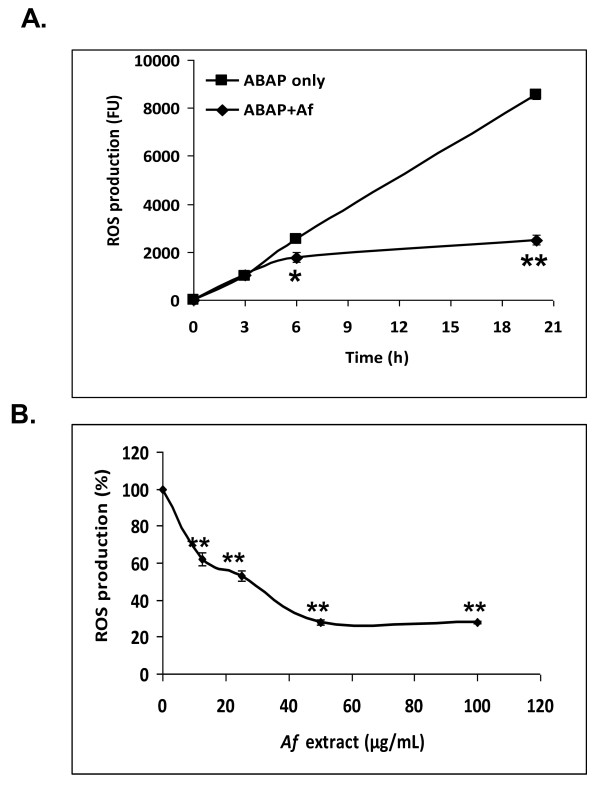
***Af *extract inhibits the peroxyl radical - induced oxidation of DCFH in primary microglial cells**. Microglial cells were incubated for 1 h with *Af *extract. They were then preloaded with DCF-DA for 30 min and washed with PBS, after which, 0.6 mM ABAP was added and ROS levels were measured at the indicated time points. Each point represents mean ± SEM of 2 experiments (n = 8). A. *Af *extract at 50 μg/mL. B. ROS production was measured 22 h after the addition of ABAP. * *p *< 0.05; ** *p *< 0.001.

The cellular antioxidant activity assay was used in order to measure the ability of compounds present in the *Af *extract to enter the cells and prevent the formation of DCF by ABAP-generated peroxyl radicals [34]. In this assay, the efficiency of cellular uptake, combined with the radical-scavenging activity dictates the efficacy of the tested compounds. The kinetics of DCFH oxidation in microglial cells by peroxyl radicals generated from ABAP is shown in Figure 4A, where it can be seen that ABAP generated radicals in a time-dependent manner, and that treatment of cells with *Af *extract moderated this induction. Figure 4B shows that the increase in ROS-induced fluorescence was inhibited by *Af *extract in a dose-dependent manner. This indicates that compounds present in the *Af *extract entered the cells and acted as efficient intracellular hydroperoxyl radical scavengers.

#### Inhibition of LPS-Induced iNOS and COX-2 Expression by the *Af* Extract

**Figure 5:**
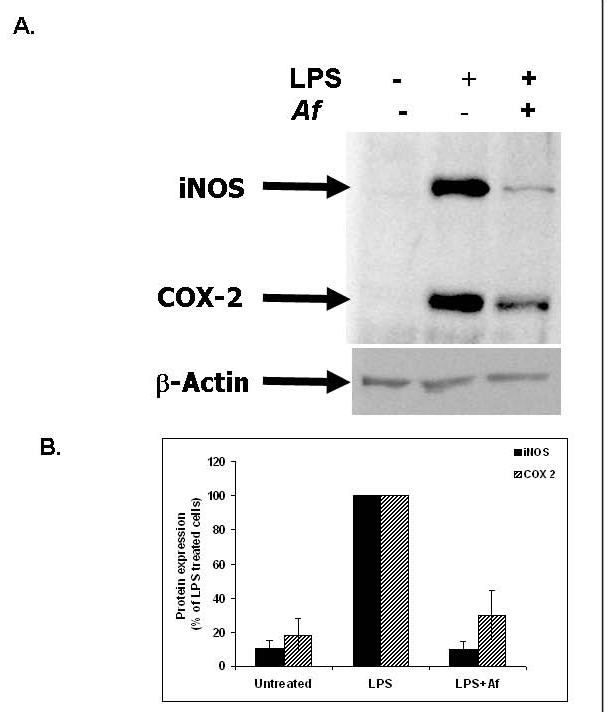
**Inhibition of iNOS and COX-2 expression in LPS-stimulated microglial cells by *Af *extract**. Microglial cells were treated with 100 μg/mL of *Af *extract, followed by stimulation with LPS for 20 h. Equal amounts of cell lysates were separated by SDS-PAGE and immunoblotted with antibodies to iNOS, COX-2, and β-actin. A. The immunoblot represents one of three different experiments with similar results. B. Data represent the means ± SEM of three independent experiments (n = 3). The levels of each protein were normalized to the levels of β-actin protein.

Cells were activated with LPS in the presence or absence of *Af *extract. Twenty hours later, cells were harvested and levels of iNOS and COX-2 were determined by Western blot analysis. While the expression levels of iNOS and COX-2 proteins were barely detectable in untreated control cells, they were markedly increased in response to LPS. Treatment with *Af *extract markedly inhibited the LPS-elicited iNOS and COX-2 expression in microglial cells (Figure 5). Expression of the internal control, β-actin, was not affected by the different treatments (Figure 5A).

#### Attenuation by *Af* Extract of IL-1β Transcription and TNFα Secretion in LPS-Stimulated Microglial Cells

**Figure 6:**
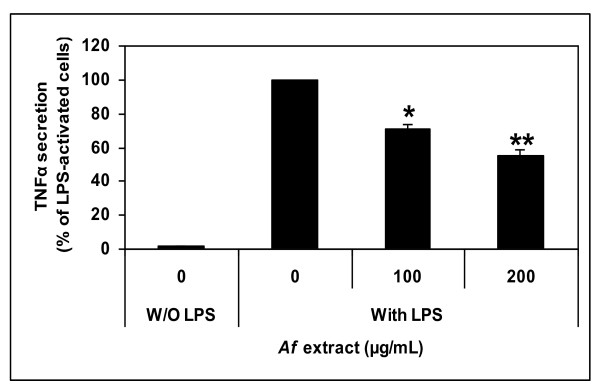
**Down-regulation of TNFα secretion from activated microglial cells by *Af *extract**. Microglial cells were treated with the indicated concentrations of *Af *extract, followed by stimulation with LPS (100 ng/mL). After 5 h, conditioned media were collected and tested for cytokine levels by ELISA. TNFα levels in the activated cells (designated as 100%) were 900 pg/mL. Data represent the means ± SEM of two independent experiments (n = 4). * *p *< 0.01; ** *p *< 0.001.

**Figure 7:**
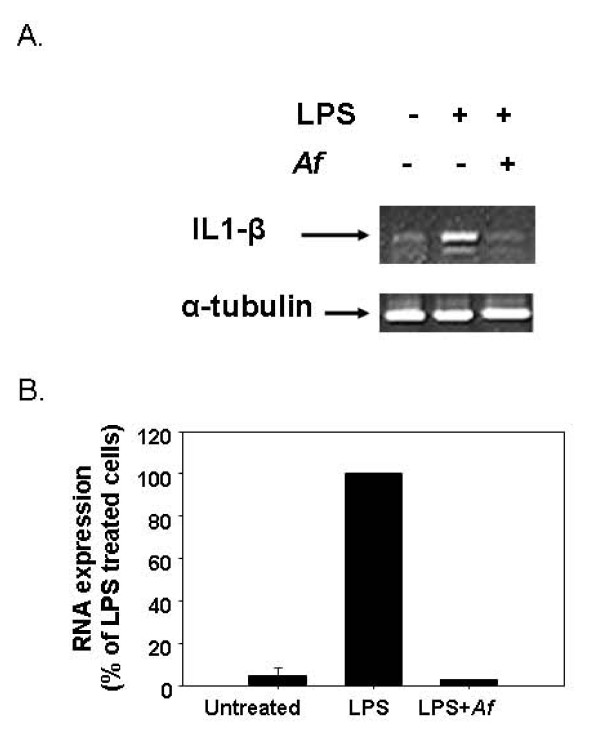
**Down-regulation of IL-1β expression by LPS-stimulated microglial cells by *Af *extract**. Microglial cells (5 × 10^6 ^cells) were treated with 100 μg/mL of *Af *extract, followed by stimulation with LPS (4.5 ng/mL) for 20 h. The products following RT-PCR were separated on agarose gel. A. The gel represents one of three different experiments with similar results. The levels of β-actin transcripts were similar in all samples. B. Data represent the means ± SD of three independent experiments (n = 3). The levels of IL-1β transcripts were normalized to the levels of α-Tubulin transcripts.

To test whether the *Af *extract reduced the release of the inflammatory cytokines TNFα and IL-1β from microglial cells, LPS was added to the culture media of the cells in the presence or absence of the *Af *extract. In unstimulated microglial cells, only a small amount of TNFα could be detected in the medium conditioned by the cells (Figure 6). However, stimulation of the cells with LPS resulted in a remarkable increase in TNFα release, which was reduced (50%) by the *Af *extract in a dose-dependent manner (Figure 6). Similarly, IL-1β transcription that had been induced by LPS in the activated microglial cells was significantly inhibited when cell activation was performed in the presence of *Af *extract (Figure 7).

#### Attenuation by *Af* Extract of MMP-9 activity in LPS-Stimulated Microglial Cells

To study the effect of the *Af *extract on MMP-9 activity in LPS-activated microglial cells, LPS was added to the culture media of microglial cells in the presence or absence of the *Af *extract, and the media conditioned by the cells was collected after 24 hr. As MMP-9 degrades denatured collagen (gelatin) in addition to collagen, MMP-9 activity was measured using gelatin zymography. As shown in Figure 3, MMP-9 activity in unstimulated microglial cells is very low. However, stimulation of the cells with LPS resulted in a remarkable increase in MMP-9 activity compared to control cells. MMP-9 activity was markedly reduced by the *Af *extract in a dose-dependent manner (Figure 3). The reduction was not a result of cell death as no toxicity was observed using the crystal violet assay for cell viability (data not shown).

## Discussion

The main findings of this study are that out of the 66 desert plant extracts which were tested, the extract of *Achillea fragrantissima *was the most active extract, and inhibited 70% of the NO produced by the activated cells. This reduction was dose dependent and did not result from a cytotoxic effect of the extract. In addition, *Af *extract inhibited the LPS-elicited expression of the proinflammatory cytokines IL-1β and TNFα and of the proinflammatory enzymes COX-2, iNOS and MMP-9 and down-regulated NO and ROS production from primary cultures of activated microglial cells. This inhibition did not result from a cytotoxic effect of the extract.

It seems that the *Af *extract is a polyvalent cocktail which contains compounds that interferes with the LPS signal as well as compounds with radical-scavenging activity that can enter the cells and react with ROS intracellularly.

Previous studies have shown that there is a complex relationship between the various anti-inflammatory compounds tested in this study; for example, activation of iNOS and COX-2 via TNFα and IL-1β stimulate the coupled release of NO and PGE_2_, while NO modulates the TNFα- and IL-1β-dependent elevation of PGE_2 _levels in astrocytes [35]. In addition, the expression of MMPs is regulated, amongst others, by inflammatory cytokines [36]. Moreover, S-nitrosylation [37] and tyrosine nitration [38] activates MMP-9 and NO is known to stimulate the enzymatic activity of COX-2 both *in vitro *[39] and *in vivo *[40].

The expression of the inflammatory molecules TNFα, IL-1β, iNOS, COX-2 and MMP-9 can be regulated through the activation of NF-κB by activators such as LPS and IL-1β [41,42]. Therefore, the inhibitory effect of the *Af *extract on the expression of these molecules might be attributed to inhibition of NF-κB activation or to other signaling events leading to the production of proinflammatory molecules in microglial cells such as protein kinase C (PKC) [43], p38 mitogen-activated protein kinase (MAPK) or p42/44 MAPK [41,44,45].

The proinflammatory molecules tested in this research are produced, not only by activated microglial cells but also by activated macrophages and many other cell types. Thus, the *Af *extract might also be beneficial in many other inflammatory diseases that are not related to neurodegenerative disease. Also, MMPs and ROS have been shown to be involved in blood brain barrier breakdown and in brain damage in bacterial meningitis [19,20].

The importance of all of these proteins in the neuroinflammatory response in various animal models of brain pathologies was demonstrated by specific inhibitors and knockout strategies of the relevant genes that could protect against brain damage in experimental pathology [18,46-49].

Thus, COX-2, iNOS and MMP-9 activities, as well as TNFα, IL-1β and NO generation have become accepted as markers and therapeutic targets in neurodegenerative diseases, and thus their down-regulation might assist in preventing or delaying the onset of these diseases.

To the best of our knowledge, the effects of *Af *in the context of neurodegenerative diseases have not been studied in the past, and this is the first study characterizing the anti-neuroinflammatory activities of this plant.

## Conclusions

On the basis of the current results, we suggest that various compounds present in the *Af *extract might have complementary beneficial bioactivities, and thus propose that *Af *extracts should be further studied as polyvalent cocktails for nutraceutical development for the prevention or treatment of neurodegenerative diseases.

## List of Abbreviations

*Af: Achillea fragrantissima*; COX: Cyclooxygenase; IL-1β: Interleukin 1 beta; iNOS: inducible nitric oxide synthase; LDH: Lactate dehydrogenase; LPS: Lipopolysaccharide; MMP: matrix metalloproteinases; NO: Nitric oxide; TNFα: Tumor necrosis factor alpha

## Competing interests

The authors declare that they have no competing interests.

## Authors' contributions

AE carried out the study design, some of the experiments, literature search and manuscript preparation. SM, HE, AT and MR carried out the cell culture and biochemical experiments. RO collected the plants, prepared the extracts, performed the RT-PCR experiments, and contributed in drafting the manuscript. All authors read and approved the final manuscript.

## Pre-publication history

The pre-publication history for this paper can be accessed here:

http://www.biomedcentral.com/1472-6882/11/98/prepub
